# Manganese-Enhanced cardiac MRI (MEMRI) tracks long-term *in vivo* survival and restorative benefit of transplanted human Amnion-Derived Mesenchymal Stem Cells (hAMSC) after porcine ischemia-reperfusion injury

**DOI:** 10.1186/1532-429X-15-S1-O106

**Published:** 2013-01-30

**Authors:** Rajesh Dash, Paul J Kim, Yuka Matsuura, Xiaohu Ge, Fumiaki Ikeno, Jennifer K Lyons, Ngan F Huang, Scott Metzler, Patricia Nguyen, Shahriar Heidary, Marie-Claude Parent, Tomoaki Yamamoto, John Cooke, Pilar Ruiz-Lozano, Robert C Robbins, Joseph C Wu, Michael V McConnell, Alan Yeung, Phillip Harnish, Phillip C Yang

**Affiliations:** 1 Medicine, Stanford University, Stanford, CA, USA; 2 Cardiac Surgery, Stanford University, Stanford, CA, USA; 3 Radiology, Stanford University, Stanford, CA, USA; 4 Pediatrics, Stanford University, Stanford, CA, USA; 5 Engineering, Stanford University, Stanford, CA, USA; 6 Eagle Vision Pharmaceutical Corp., Downington, PA, USA

## Background

It is unclear whether transplanted stem cells, despite their functional benefits, survive and engraft in the heart following transplantation. hAMSCs exhibit cell surface markers of immunomodulation (HLA-DR -, HLA-G +, CD59 +) that may enhance survival after transplantation. To investigate the viability of hAMSCs in vivo, we used a MEMRI contrast agent, EVP-1001-1 (Eagle Vision Pharmaceuticals, Inc) in a porcine ischemia-reperfusion (IR) injury model. EVP-1001-1 specifically enters live cells via L-type Ca2+ channels. Following EVP-1001-1 injection, MEMRI delineates the infarct zones through T1 signal loss. EVP-1001-1 also produces increased T1 signal in isolated hAMSCs.

## Methods

Seven adult farm pigs underwent 60 min LAD coronary IR. One week post-IR, pigs hearts were injected with either hAMSCs (~80 million cells/heart, n=4) or normal saline (NS, n=3) into ~8 peri-infarct and infarct zones, by intraventricular catheter injection (Biocardia, Inc.). Cardiac MRI (CMR) was performed serially to assess ejection fraction (EF%), infarct % by delayed gadolinium Enhancement MRI (DEMRI), and myocardial viability % (MEMRI). (DEMRI & MEMRI: GE 3T Signa Excite HD: FGRE-irP: RT 4.7 ms, TE 1.3 ms, FOV 30, TI 200-400 ms, FA 10, ST 10 mm, 222x192).

## Results

**Figure 1:**
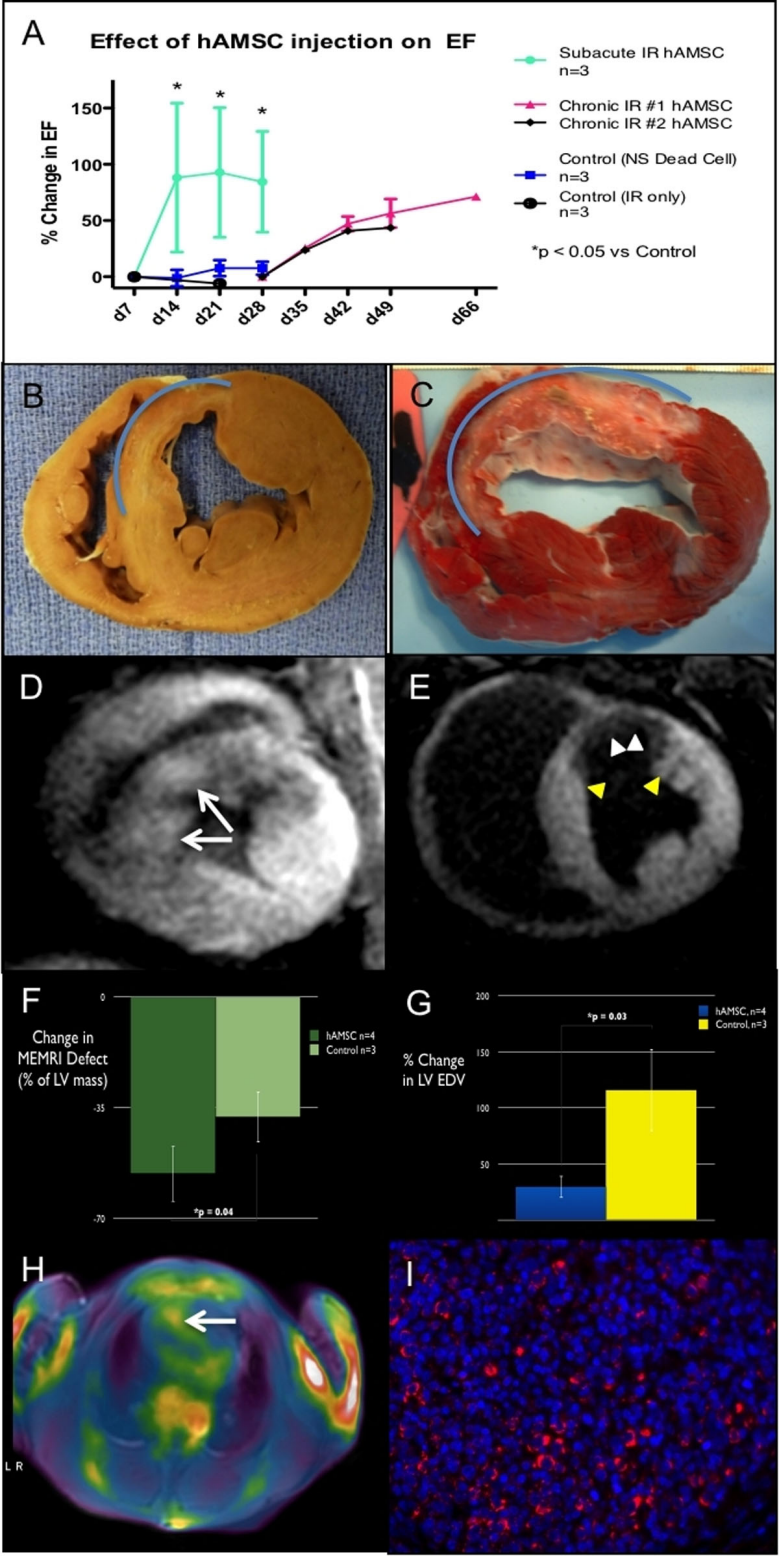


hAMSC and NS EFs were similar at baseline (57±4%, n=5) and 1wk post-IR (24±6%). However, hAMSC injection improved EFs at 1, 2, & 3wks post-hAMSC delivery, compared to NS-injected swine (Fig 1A). A possible mechanism for the improvement was increased peri-infarct viability. In support of this, MEMRI defect (infarct) volume decreased from d7 to d21 post-IR in hAMSC hearts (60±12% reduction, n=3) more than in NS hearts (38±18% reduction; Fig 1 F,G). MEMRI also identified foci of high contrast-to-noise ratio (CNR) within infarct zones in hAMSC hearts (hAMSC: 8.6±1.4*; NS: 4.9±0.8, n=3, *p<0.05 Fig 1D,1E), suggesting increased EVP-1001-1 uptake by live hAMSCs within the infarct zone. This signal also increased from d10 to d17. In two swine, 20% of the hAMSCs were transduced with a HSV-tk PET reporter gene, and cardiac PET imaging confirmed co-localizing PET and MEMRI signals (Fig 1H), indicating live stem cell populations (Fig 1I). Human anti-mitochondrial Ab immunostaining revealed viable hAMSC cell clusters in infarct zones 38 days post-transplantation.

## Conclusions

These results demonstrate that hAMSC delivery in a porcine IR model improves systolic function compared to control. The mechanism for this functional restoration may be due to improved peri-infarct viability by salvage of the injured cardiomyocytes. High MEMRI CNR within the infarct zone was associated with positive cardiac PET signal as well as hNA staining, indicating live hAMSC populations nearly 6 weeks after cell delivery. Moreover, MEMRI allows for non-invasive assessment of myocardial viability and tracking of stem cell survival in vivo, without any need for genetic pre-modification of the transplanted stem cells.

## Funding

NIH R01 (PY).

NIH, NHLBI K08 (RD).

